# Gastric cancers of Western European and African patients show different patterns of genomic instability

**DOI:** 10.1186/1755-8794-4-7

**Published:** 2011-01-13

**Authors:** Tineke E Buffart, Melanie Louw, Nicole CT van Grieken, Marianne Tijssen, Beatriz Carvalho, Bauke Ylstra, Heike Grabsch, Chris JJ Mulder, Cornelis JH van de Velde, Schalk W van der Merwe, Gerrit A Meijer

**Affiliations:** 1 Dept. of Pathology, VU University Medical Center, Amsterdam, The Netherlands; 2 Dept. of Anatomical Pathology, University of Pretoria, Pretoria, South Africa; 3 Pathology and Tumour Biology, Leeds Institute of Molecular Medicine, University of Leeds, Leeds, UK; 4 Dept. of Gastroenterology, VU University Medical Center Amsterdam, The Netherlands; 5 Dept. of Surgery, Leiden University Medical Center, Leiden, The Netherlands; 6 Dept of Internal Medicine and Gastroenterology, University of Pretoria, South Africa

## Abstract

**Background:**

Infection with *H. pylori *is important in the etiology of gastric cancer. Gastric cancer is infrequent in Africa, despite high frequencies of *H. pylori *infection, referred to as the African enigma. Variation in environmental and host factors influencing gastric cancer risk between different populations have been reported but little is known about the biological differences between gastric cancers from different geographic locations. We aim to study genomic instability patterns of gastric cancers obtained from patients from United Kingdom (UK) and South Africa (SA), in an attempt to support the African enigma hypothesis at the biological level.

**Methods:**

DNA was isolated from 67 gastric adenocarcinomas, 33 UK patients, 9 Caucasian SA patients and 25 native SA patients. Microsatellite instability and chromosomal instability were analyzed by PCR and microarray comparative genomic hybridization, respectively. Data was analyzed by supervised univariate and multivariate analyses as well as unsupervised hierarchical cluster analysis.

**Results:**

Tumors from Caucasian and native SA patients showed significantly more microsatellite instable tumors (p < 0.05). For the microsatellite stable tumors, geographical origin of the patients correlated with cluster membership, derived from unsupervised hierarchical cluster analysis (p = 0.001). Several chromosomal alterations showed significantly different frequencies in tumors from UK patients and native SA patients, but not between UK and Caucasian SA patients and between native and Caucasian SA patients.

**Conclusions:**

Gastric cancers from SA and UK patients show differences in genetic instability patterns, indicating possible different biological mechanisms in patients from different geographical origin. This is of future clinical relevance for stratification of gastric cancer therapy.

## Background

Gastric cancer is the second most common cause of cancer death worldwide, but incidence and mortality rates show large variations across different countries. Japan and China show the highest incidence rates of gastric cancer of 80-115 cancers/100,000 population and 32-59 cancers/100,000 population respectively, while in other Asian counties, such as India, Bangladesh, and Thailand, the incidence rates are much lower (10.6, 1.3, and 7.1 per 100,000 populations, respectively). Also within Europe, incidence and mortality rates differ between countries. Portugal has the highest incidence rates (33.2/100,000) whereas other countries in Western Europe show incidence rates of 19.4 per 100,000 populations. In the Netherlands it ranks fifth as a cause of cancer death with incidence rates of 14.6/100,000. In Africa, gastric cancer is infrequent, with incidence rates varying between 6.9/100,000 in Northern Africa, 12.9/100,000 in Eastern Africa, 11.9/100,000 in Southern Africa and 7.0/100,000 in Western Africa (Table 1) [1-3].

**Table 1:** Incidence rates of gastric cancers per 100,000 populations.

	Incidence rates		Incidence rates
**Japan**	80-115	**Netherlands**	14.6
**China**	32-59	**Western Europe**	19.4
**India**	10.6	**Northern Africa**	6.9
**Bangladesh**	1.3	**Eastern Africa**	12.9
**Thailand**	7.1	**Southern Africa**	11.9
**Portugal**	33.2	**Western Africa**	7.0

According to the Correa model, intestinal type gastric cancers arise through a sequence of events, starting with chronic active gastritis due to infection with *Helicobacter pylori *(*H. pylori*). This chronic inflammatory process may lead to atrophy, intestinal metaplasia followed by dysplasia and eventually may lead to invasive adenocarcinoma [4].

The mechanism by which *H. pylori *contributes to gastric carcinogenesis is still largely unknown. However, we do know that gastric cancer is the result of accumulation of (epi)genetic changes. In gastric cancer, at least two types of genetic instability play a role. Microsatellite instability (MSI) occurs in cancers associated with Lynch syndrome or hereditary non-polyposis colorectal cancer (HNPCC), and in 10-15% of sporadic gastric cancers due to *hMLH1 *promoter hypermethylation [5,6]. However, the majority of gastric cancers show chromosomal instability, resulting in DNA copy number aberrations that can be analyzed in detail by high resolution array comparative genomic hybridization (array CGH). In a previous study using chromosome based comparative genomic hybridization (CGH), we were unable to demonstrate that there are specific chromosomal alterations which are associated with *H. pylori *infection [7].

Infection with *H. pylori *is important in the etiology of gastric cancer, consequently high incidences of gastric cancer are observed in areas with high prevalence of *H. pylori *infection, like Asia. However, despite high frequencies of *H. pylori *infection in Africa, gastric cancer is infrequent in Africa, a phenomenon often referred to as the 'African enigma' [8,9]. We hypothesize that geographical differences in environmental factors, including infection with *H. pylori*, and host factors are reflected by different biological characteristics of the tumors from those areas. Therefore, we compared MSI status and DNA copy number profiles in gastric cancer patients from United Kingdom (UK) and South Africa (SA).

## Methods

### Material

A total of 67 gastric adenocarcinomas were included in this study. Of these, 33 gastric adenocarcinomas were obtained from Leeds (Leeds, General Infirmary, UK) and 34 gastric adenocarcinomas were obtained from Pretoria (Prinshof Campus, Pretoria, South Africa), of which 25 were obtained from native South African patients (native SA) and 9 from Caucasian South African patients (Caucasian SA), respectively. All tumors were randomly selected after testing for proper DNA quality as previously described [10]. All gastric adenocarcinomas were staged according to the TNM classification (5th edition) for the grading and to the Laurén's classification for morphology [11]. The study was approved by the Institutional Review Board and was in accordance with local medical ethical regulations.

### DNA isolation procedure

DNA was isolated from formalin-fixed and paraffin embedded gastric cancer material as described previously,[12,13] using the QIAamp microkit (Qiagen, Hilden, Germany). DNA concentrations were measured using a Nanodrop ND-1000 spectrophotometer (Isogen, IJsselstein, The Netherlands) and DNA quality was assessed by isothermal amplification [10]. Genomic DNA isolated from peripheral blood obtained from eighteen healthy females or males was pooled to use as normal reference.

### Microsatellite instability (MSI) analysis

MSI analysis was performed using the MSI Analysis System (MSI Multiplex System Version 1.1, Promega) consisting of five nearly monomorphic mononucleotide markers *(BAT-25, BAT-26, NR-21, NR-24, MONO-27) *according to the manufacturer's instructions. PCR products were separated by capillary electrophoresis using an ABI 3130 DNA sequencer (Applied Biosystems, Foster City, CA, USA), and analyzed using GeneScan 3100 (Applied Biosystems, Foster City, CA, USA). An internal lane size standard was added to the PCR samples for accurate sizing of alleles and to adjust for run-to run variations. When all markers were stable, the tumor was interpreted as microsatellite stable (MSS). The tumor was interpreted as MSI-low (MSI-L) if one marker was instable and MSI-high (MSI-H) if two or more markers showed instability. MSI-L tumors were included in the MSS category in further analysis. Due to polymorphisms[14] in the South African population, native South African tumors were classified as MSI when three or more markers were instable.

### Array CGH

Array CGH was performed as described before [12,15]. Briefly, 600 ng tumor and normal reference DNAs were labeled by random priming (Bioprime DNA Labeling System, Invitrogen, Breda, The Netherlands) and hybridized onto a BAC array containing approximately 6000 clones, consisting of the Sanger BAC clone set with an average resolution along the whole genome of 1.0 Mb, the OncoBac set, containing approximately 600 clones corresponding to 200 cancer-related genes, and selected clones of interest obtained from the Children's Hospital Oakland Research Institute (CHORI) to fill gaps larger than 1 Mb on chromosome 6 and to have full coverage contigs of regions on chromosome 8, 13 and 20. All clones were printed in triplicate on Nexterion slides (Schott Nexterion, Jena, Germany). Subsequent analysis was performed according to the clone position from the UCSC May 2004 freeze of the Human Genome Golden Path http://genome.ucsc.edu.

### Image acquisition and data analysis

Images of the arrays were acquired by scanning (Agilent DNA Microarray scanner, Agilent Technologies, Palo Alto, USA) and Bluefuse software version 3.4 (BlueGnome, Cambridge, UK) was used for automatic feature extraction. Spots were excluded when the quality flag was below 1 or the confidence value was below 0.1. Log_2 _tumor to normal ratio was calculated for each clone and median block normalization was used to normalize the data. Quality of array CGH profiles was measured by calculating a median absolute deviation value of chromosome 2 (MAD2) [10]. Array CGH profiles with MAD2 values >0.18 were excluded from further analysis. For determining copy number gains and losses, the R package CGH call was used [16]. Output of the CGH call analysis was used for CGH region analysis to compress the data, using a threshold for average error rate of 0.001 [17]. Hierarchical cluster analysis was performed using the WECCA program, with the parameter total linkage [18].

Array data can be accessed using the Gene Expression Omnibus (GEO) http://www.ncbi.nlm.nih.gov/geo/, under accession number GSE22789.

### Statistical analysis

Significance of differences for categorical variables between different categories was tested using a chi-square test. One-way ANOVA with Bonferroni correction was used to calculate significant differences for continuous variables between Caucasian SA, native SA, and UK patients (SPSS 12.0.1 for Windows, SPSS Inc, Chicago, IL, USA). P values less than 0.05 were considered to be significant.

Supervised analysis was performed using the non-parametric Mann-Whitney two-sample test (CGH test [19]). Alterations in patterns between different tumor groups were compared using a binomial differential proportion test. The test procedure included a permutation-based false discovery rate correction for multiple testing [20]. Two-sided p values less than 0.05 and false discovery rates below 0.15 were considered to be significant.

## Results

### Clinicopathological data

The mean age of the UK gastric cancer patients was 73.3 years (range 51-96), mean age of the Caucasian SA patients was 68.0 years (range 56-84) and the mean age of the native SA patients was 56.5 years (range 29-79). One-way ANOVA with Bonferroni correction yielded a significant difference between the mean age of the patients between native and Caucasian SA patients (p = 0.03) and between native SA and UK patients (p < 0.001), but not between Caucasian SA and UK patients (n.s).

There was no significant difference between patients of different geographical location and gender, tumor stage (T-category) and lymph node stage (N-category). UK gastric cancers showed significantly more diffuse type morphology compared to South African gastric cancers (p = 0.002). Overview of patient and tumor characteristics is given in Table 2.

**Table 2:** Tumor and patient characteristics of the 67 tumors used for MSI and array CGH analysis.

ID	gender	age	Tumor type	T	N	origin	MSI status	Cluster number	Cluster order	% events	% gains	% losses
1	F	62	intestinal	T2	N1	Cauc SA	MSS	5	34	3.2	3.2	0
2	M	73	intestinal	T2	N2	Cauc SA	MSS	5	28	16.9	13.4	3.4
3	F	59	intestinal	T2	N1	Cauc SA	MSS	5	26	26.5	15.1	11.4
4	F	74	intestinal	T2	N1	Cauc SA	MSS	5	27	15.7	9.3	6.5
5	M	56	intestinal	T3	N1	Cauc SA	MSI	-	-	15.6	10.3	5.3
6	M	57	intestinal	T2	N1	Cauc SA	MSS	6	48	3.4	2.4	1.0
7	F	84	intestinal	T1	N0	Cauc SA	MSS	6	43	10.9	7.9	3.1
8	F	79	intestinal	T3	N0	Cauc SA	MSI	-	-	17.6	16.8	0.8
9	M	68	intestinal	T3	N2	Cauc SA	MSS	5	25	32.1	24.3	7.7
10	M	65	intestinal	T2	N0	native SA	MSI	-	-	1.7	1.7	0
11	M	57	intestinal	T1	N0	native SA	MSI	-	-	0	0	0
12	F	29	intestinal	T4	N0	native SA	MSS	6	37	28.3	26.2	2.1
13	F	59	intestinal	T3	N1	native SA	MSS	6	42	10.6	5.3	5.3
14	M	66	intestinal	T2	N1	native SA	MSS	5	31	6.4	6.4	0
15	M	46	intestinal	T3	N2	native SA	MSS	5	35	3.2	3.2	0
16	F	-	diffuse	T4	N2	native SA	MSS	6	44	13.2	12.4	0.7
17	M	51	intestinal	T3	N1	native SA	MSS	5	32	6.1	6.1	0
18	F	49	intestinal	T3	N1	native SA	MSS	4	20	29.1	18.2	10.9
19	M	56	intestinal	T3	N1	native SA	MSS	4	19	26.0	10.3	15.7
20	M	48	intestinal	T3	N2	native SA	MSS	5	36	5.4	5.4	0
21	M	65	mixed	T3	N1	native SA	MSI	-	-	18.2	16.3	1.9
22	M	60	intestinal	T2	-	native SA	MSS	6	41	14.0	11.3	2.7
23	F	63	intestinal	-	-	native SA	MSS	6	38	17.1	10.7	6.4
24	F	54	papillary	T2	N0	native SA	MSS	3	14	44.8	20.6	24.2
25	M	67	intestinal	T3	N1	native SA	MSS	-	-	-	-	-
26	M	31	intestinal	T3	N1	native SA	MSS	5	24	41.1	28.4	12.7
27	M	43	intestinal	T3	N1	native SA	MSI	-	-	9.9	9.9	0
28	F	71	intestinal	T3	-	native SA	MSS	5	33	3.7	2.7	1.0
29	F	77	intestinal	T3	N1	native SA	MSS	5	30	12.7	7.1	5.5
30	M	57	intestinal	T2	N0	native SA	MSS	-	-	-	-	-
31	M	79	intestinal	T3	N2	native SA	MSI	-	-	2.5	2.5	0
32	M	57	intestinal	T4	N0	native SA	MSI	-	-	17.8	16.8	1.0
33	M	56	mixed	T3	N1	native SA	MSS	4	17	42.2	21.3	20.9
34	F	49	mixed	T3	N3	native SA	MSS	5	29	22.3	19.2	3.2
35	F	82	diffuse	T1	N0	UK	MSS	6	46	11.2	10.3	1.0
36	M	81	diffuse	T3	N2	UK	MSS	4	22	15.7	7.2	8.5
37	M	71	diffuse	T2	N1	UK	MSS	7	53	18.4	14.7	3.7
38	M	73	intestinal	T2	N0	UK	MSS	2	11	26.0	17.2	8.8
39	F	65	diffuse	T2	N3	UK	MSS	6	45	15.0	12.0	3.1
40	F	58	diffuse	T3	N3	UK	MSS	1	6	25.7	13.7	12.0
41	F	51	diffuse	T3	N3	UK	MSS	1	7	22.4	16.5	6.0
42	M	91	intestinal	T1	N0	UK	MSS	7	56	49.2	27.6	21.6
43	M	71	diffuse	T3	N2	UK	MSS	7	49	40.6	19.8	20.8
44	M	73	intestinal	T2	N1	UK	MSS	1	5	21.0	8.2	12.9
45	M	64	diffuse	T2	N0	UK	MSS	7	50	14.9	10.3	4.6
46	F	71	intestinal	T1	N0	UK	MSS	1	4	40.8	13.4	27.4
47	F	60	intestinal	T4	N1	UK	MSS	3	16	30.1	15.6	14.5
48	F	96	diffuse	T3	N0	UK	MSS	2	12	41.0	25.0	16.0
49	M	91	mixed	T3	N0	UK	MSS	6	40	16.0	11.4	4.6
50	M	81	diffuse	T2	N0	UK	MSS	2	10	37.7	17.2	20.4
51	F	83	intestinal	T2	N0	UK	MSS	7	51	14.5	10.5	4.0
52	F	82	intestinal	T3	N1	UK	MSS	6	47	2.2	2.2	0
53	F	77	intestinal	T3	N1	UK	MSS	6	39	19.5	11.1	8.5
54	M	74	mixed	T2	N1	UK	MSS	2	8	44.8	22.4	22.3
55	F	59	diffuse	T3	N1	UK	MSS	3	15	26.4	18.1	8.2
56	M	77	mixed	T3	N2	UK	MSS	1	3	24.1	13.7	10.3
57	M	75	intestinal	T2	N0	UK	MSS	2	9	36.9	18.2	18.7
58	M	64	diffuse	T3	N3	UK	MSS	2	13	35.3	20.2	15.1
59	M	71	intestinal	T3	N1	UK	MSS	1	2	31.0	11.7	19.3
60	F	74	diffuse	T3	N2	UK	MSS	4	23	23.2	14.7	8.4
61	M	81	intestinal	T2	N0	UK	MSI	-	-	10.7	8.7	2.0
62	F	74	mixed	T3	N2	UK	MSS	4	18	30.4	17.9	12.5
63	M	67	mixed	T3	N1	UK	MSS	7	52	12.3	6.4	5.9
64	M	73	intestinal	T3	N1	UK	MSS	4	21	15.2	7.3	7.9
65	F	66	mixed	T3	N2	UK	MSS	7	55	34.3	18.7	15.6
66	M	82	intestinal	T1	N1	UK	MSS	7	54	46.7	27.4	19.3
67	F	62	mixed	T3	N0	UK	MSS	1	1	44.0	20.7	23.3

Percentages of events, gains and losses are given for all tumors of sufficient array CGH quality. Cluster number and order are listed for the 56 cases included in the cluster analysis.

F: female, M: male, T: T-stage, N: N-stage MSS: microsatellite stable, MSI: microsatellite instable, Cauc SA: Caucasian South African patients, native SA: native South African patients, UK: patients from United Kingdom, -: unknown.

### Microsatellite instability (MSI) analysis

Two out of nine (22%) Caucasian SA gastric cancers, six out of 25 (24%) native SA gastric cancers, and one out of 33 (3%) UK gastric cancers showed MSI. All other gastric cancers were MSS (Table 2). Pearson chi-square yielded a significant difference between the three different tumor groups and MSI status (p < 0.05).

### Hierarchical cluster analysis

We analyzed DNA from all gastric cancers by genome-wide array CGH analysis to unravel DNA copy number changes in tumors from different geographical location. MSI positive gastric cancers and gastric cancers with array CGH profiles with a MAD2 value above 0.18 were excluded for cluster analysis leaving 56 tumors (from 32 UK, 17 native SA and 7 Caucasian SA patients) for further analysis.

**Figure 1:**
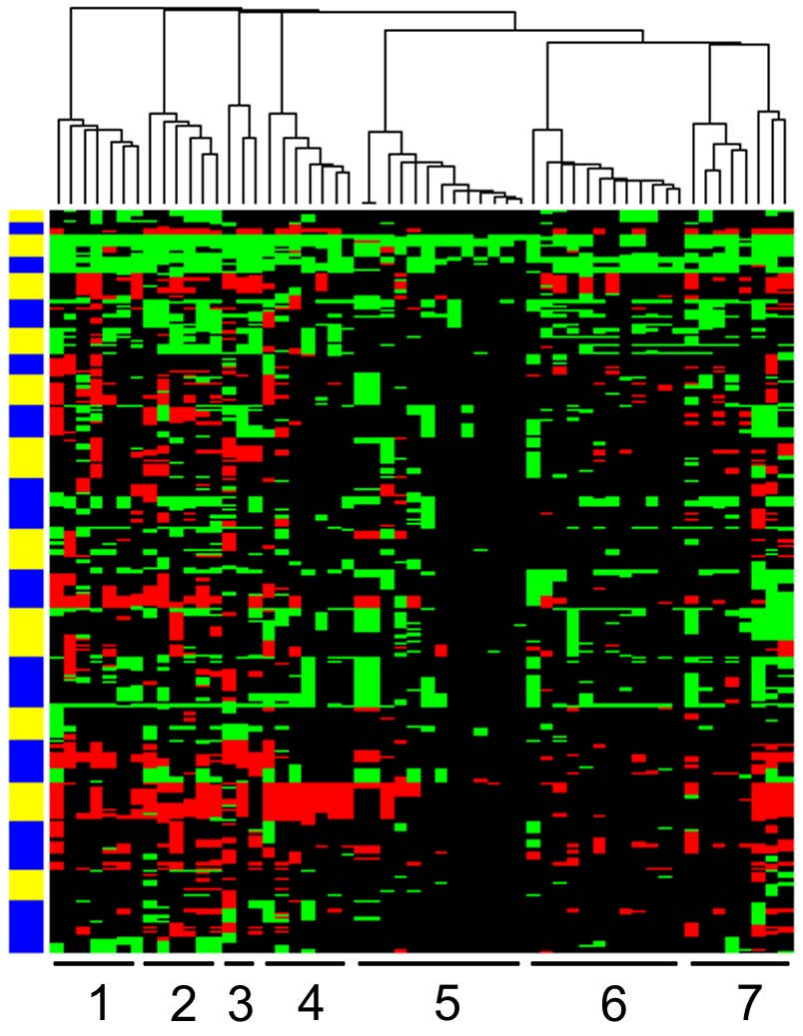
**Cluster analysis of 56 gastric adenocarcinomas of which 32, 17 and 7 were obtained from UK, native SA and Caucasian SA patients, respectively**. Hierarchical cluster analysis yielded 7 clusters significantly correlated with geographical origin of the tumors (p < 0.001). Columns represent the different tumors and rows represent the different chromosomal regions, with chromosome 1 at the bottom and chromosome 22 at the top of the heatmap. DNA copy number gains and losses are indicated in green and red, respectively. The yellow and blue bar next to the cluster represents the chromosome separation.

Hierarchical cluster analysis yielded seven clusters which were significantly correlated with gastric cancers of different geographical origin (p < 0.001) (Figure 1). Clusters 1, 2 and 7 obtained only gastric cancers from UK patients. Clusters 3 and 4 comprised of gastric cancers from UK and native SA patients. Cluster 5 contained only gastric cancers from SA patients, and cluster 6 contained a mixture of tumors of all three groups (Table 2).

UK patients showed significantly more gastric adenocarcinomas of the diffuse type according to the Laurén classification[11] compared to SA patients (p = 0.002). We therefore repeated the cluster analysis including only intestinal type gastric carcinomas. Cluster membership of the remaining 12 tumors from UK patients and 12 and 7 tumors from native SA and Caucasian SA patients, respectively, was again significantly correlated to geographical origin of the patient (p < 0.001). Moreover, when analyzing only UK gastric cancers, hierarchical cluster analysis did not separate intestinal and diffuse type gastric cancers, nor were any significant differences observed between these two morphological tumor types with supervised analysis using CGH test.

Cluster membership was independent of gender, tumor stage, lymph node stage and of age of the patients (categorized as < 50 years of age *versu*s ≥ 50 years of age).

### DNA copy number changes

We first compared the number of events, which was defined as percentage of clones showing a gain or loss. Gastric cancers from UK patients showed a higher number of events (27% (range 2-49%)) compared to cancers from Caucasian SA (16% (range 3-32%)) and native SA patients (16% (range 0-45%)) (p = 0.005). Cancers from UK, Causasian SA and native SA patients showed 15% (range 2-28%), 11% (range 2-24%) and 11% (range 0-28%) of gained clones respectively, and 12% (range 0-27%), 4% (range 0-11%) and 5% (0-24%) clones showing a loss, respectively. A significant difference in the percentage of clones showing a loss was observed between UK patients and Caucasian SA patients (p = 0.002) and between UK patients and native SA patients (p = 0.02).

Also, when looking only at microsatellite stable gastric cancers UK patients showed a higher number of events (27% (range 2-49%)) compared to microsatellite stable cancers from Caucasian SA and native SA patients (16% (range 3-32%) and 19% (range 3-45%), respectively; p = 0.04). Microsatellite stable cancers from UK, Caucasian SA and native SA patients showed 15% (range 2-28%), 12% (range 2-24%) and 13% (range 3-28%) of gained clones, respectively. There was again a significant difference in percentages of clones showing a loss between cancers from UK and Caucasian SA patients (12% (range 0-27%) and 4% (range 0-11%), respectively; p = 0.04) and between cancers from UK and native SA patients (12% (range 0-27%) and 7% (range 0-24%) respectively; p = 0.04).

**Figure 2:**
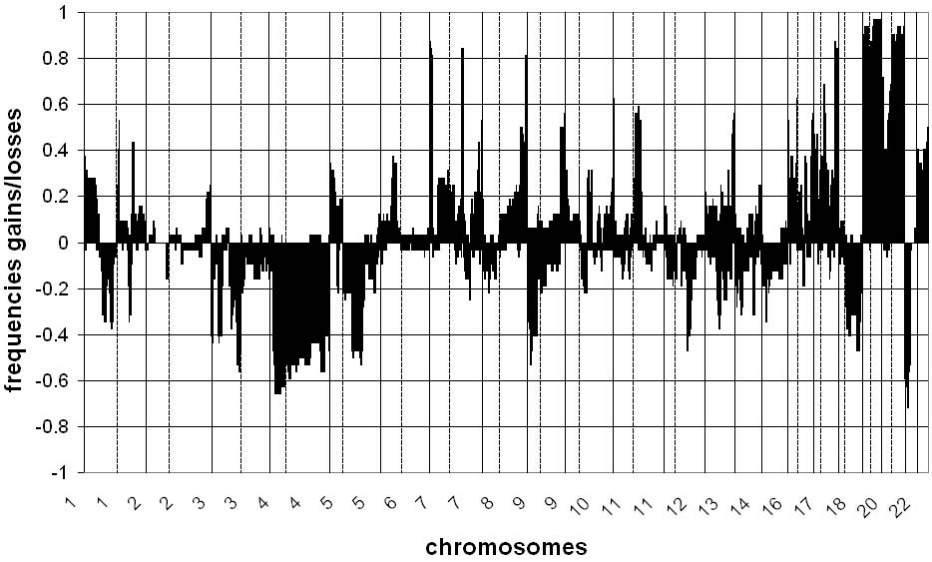
**Frequencies of gains and losses throughout the genome in all gastric adenocarcinomas from UK patients**. Clones are sorted by position per chromosome (1-22). Vertical lines indicate transition between chromosomes; dashed vertical lines indicate centromere position.

An overview of frequently altered (>30%) chromosomal regions with gains and losses per tumor group is given in Tables 3, 4 and 5. Most frequently altered (>30%) chromosomal regions observed in the UK tumors were gains on chromosomes 1p, 1q, 5p, 6p, 7p, 7q, 8q, 9q, 10p, 10q, 11p, 11q, 13q, 14q, 16p, 16q, 17p, 17q, 19p, 19q, 20p, 20q, 21q and 22q and losses on chromosomes 1p, 1q, 3p, 4p, 4q, 5q, 9p, 12q, 13q, 14q, 15q, 17q, 18q and 21q (Table 3). Most frequent DNA copy number aberrations in the native SA patients were gains on the chromosomal regions 7p, 7q, 8q, 9q, 17q, 19p, 19q, 20p and 20q, and losses on 3p and 4q (Table 4). Most frequently altered chromosomal regions in Caucasian SA patients were gains on 3q, 5p, 7p, 7q, 8p, 8q, 9q, 11q, 16p, 17q, 19p, 19q, 20p and 20q and losses on 3p, 4q and 9p (Table 5). A summary of frequencies of gains and losses of all gastric cancers per tumor group is presented in Figures 2 (UK), 3 (native SA) and 4 (Caucasian SA).

**Table 3:** Detailed overview of frequent DNA copy number aberrations (>30%) of tumors from UK patients.

chromosomal aberrations		flanking clones		position (bp)		segment size
gains	losses	start	end	start	end	(Mb)
1p36.33-p36.21		RP11-206L10	RP4-636F13	672780	12417597	11.74
	1p31.1	RP5-944F13	RP11-246O4	69815162	83112098	13.30
	1p21.3-p13.3	RP11-146P11	RP5-1077K16	95695632	107389118	11.69
1q21.2-q23.1		RP4-790G17	RP11-214H6	146971278	153444622	6.47
	1q31.1-q31.3	RP11-134C1	RP11-75C23	184717073	194242465	9.53
1q32.1-q32.2		RP11-150l7	RP11-564A8	197877387	203602276	5.72
	3p26.3	RP11-385A18	RP11-129K1	46140	2377366	2.33
	3p25.1-p24.1	RP11-255O19	RP11-99M10	15780361	30799547	15.02
	3p14.2	RP11-170K19	RP11-114P15	59701329	62639806	2.94
	3p12.3-p11.2	RP11-103P13	RP11-91M15	75146113	96627928	21.48
	4p16.1-q35.2	RP11-61G19	CTC-963K6	10275012	191158370	180.88
5p15.33		RP11-811I15	CTD-2265D9	70262	2671745	2.60
	5q11.1-q23.3	RP11-269M20	RP11-114H7	49913067	130460728	80.55
6p21.32-p21.1		RP11-79I1	RP11-121G20	33123932	44385866	11.26
7p22.3-p22.1		RP11-713A20	RP11-161C7	106471	6396697	6.29
7p11.2		RP11-449G3	RP11-34J24	54413814	55403627	0.99
7q22.1		RP11-10D8	RP11-163M5	98067793	101528379	3.46
7q36.1		RP11-89P11	RP11-43l19	147485335	151131938	3.65
7q36.3		RP11-58F7	RP11-120H14	157072238	158524109	1.45
8q24.12-q24.3		RP11-22A24	RP5-1109M23	120711365	146238749	25.53
	9p24.3-p21.1	RP11-48M17	RP11-141J7	2136329	32469400	30.33
9q33.3-q34.3		RP11-205K6	RP11-424E7	126296075	138363252	12.07
10p15.3		RP11-631M21	RP11-74N14	50000	1789100	1.74
10p15.2		RP11-195B3		3293007	3338470	0.05
10q22.1		RP11-91A1	RP11-28E3	72033907	73573433	1.54
11p15.5-p15.4		CTC-908H22	RP11-304P12	178227	3140168	2.96
11q12.2-q13.5		RP11-286N22	RP11-30J7	60851860	76232373	15.38
	12q21.2-q22	RP1-97G4	RP11-2K12	76228586	91346299	15.12
13q11-q12.11		RP11-94A1	RP11-61K9	18360157	19386914	1.03
	13q21.2-q21.33	RP11-310K10	RP11-451E2A	60721181	71574154	10.85
13q32.3		RP11-19J14	RP11-113F15	97851594	99328275	1.48
13q33.3-q34		RP11-61I17	RP11-569D9	108847725	114103243	5.26
	14q12	RP11-330O19	RP11-109D12	25538832	26345513	0.81
	14q21.1-q21.3	RP11-88D14	RP11-94K16	36949212	48298851	11.35
	14q31.1-q31.3	RP11-46l17	RP11-88N20	78630860	86772926	8.14
14q32.31-q32.33		RP11-367F11	RP11-815P21	101467534	105159201	3.69
	15q14	RP11-294M6	RP11-79A5	33841939	35953471	2.11
16p13.3		CTD-2148K8	RP11-89M4	87754	4697230	4.61
16p13.2-p13.11		RP11-475D10	RP11-489O1	8598165	15572359	6.97
16p12.1-p11.2		RP11-142A12	RP11-18H23	26595069	31443695	4.85
16q21-q22.1		RP11-52B24	RP11-394B2	63708677	69365102	5.66
16q23.3-q24.3		RP11-483P21	RP11-566K11	82361609	88613383	6.25
17p13.3-p13.1		RP11-411G7	RP11-89A15	427024	8365794	7.94
17p11.2		RP11-524F11	RP1-162E17	17343389	19251691	1.91
17q11.2-q21.31		RP11-138P22	RP11-374N3	23133763	41096064	17.96
17q21.32-q21.33		RP11-234J24	RP11-506D12	42655422	46333070	3.68
	17q22-q23.2	RP11-143M4	RP11-139B3	47607556	51363278	3.76
17q24.3-q25.3		RP11-65C22	RP11-258N23	68165339	78308832	10.14
	18q11.2-q23	RP11-5G23	RP11-396D4	21431314	71337306	49.91
19p13.3-q13.43		RP11-110A24	GS1-1129C9	134914	63771717	63.64
20p13-q13.33		RP11-530N10	CTB-81F12	9943	62393015	62.38
	21q11.2-q22.11	RP11-72P4	RP11-41N19	13857799	30673984	16.82
22q11.1-q11.21		RP11-81H21	RP11-586I18	14754982	18976359	4.22
22q12.3-q13.33		RP11-90I17	CTB-99K24	35686144	49397088	13.71

**Table 4:** Detailed overview of frequent DNA copy number aberrations (>30%) of tumors from native SA patients.

chromosomal aberrations		flanking clones		position (bp)		segment size
gains	losses	start	end	start	end	(Mb)
	3p14.2	RP11-734E15	RP11-137N22	59105371	61252524	2.15
	4q35.2	RP11-354H17	CTC-963K6	190095484	191158370	1.06
7p22.3-p11.2		RP11-713A20	RP11-80l24	106471	55784518	55.68
7q22.1		RP11-10D8	RP11-163M5	98067793	101528379	3.46
8q24.3		RP11-472K18	RP5-1109M23	144481535	146238749	1.76
9q33.3-q34.3		RP11-91G7	RP11-424E7	124316484	138363252	14.05
17q12-q21.31		RP11-893G17	RP11-392O1	31506328	39091575	7.59
17q21.32-q21.33		RP1-62O9	RP11-506D12	44647598	46333070	1.69
17q23.2-q25.3		RP11-579A4	RP11-258N23	54149948	78451750	24.30
19p13.3		RP11-110A24	CTC-1482H14	134914	5154803	5.02
19p13.2-p13.11		RP11-197O4	RP11-88I12	10248852	19023254	8.77
19q12-q13.34		CTC-1459F4	GS1-1129C9	32889410	63771717	30.88
20p13-q13.33		RP11-530N10	CTB-81F12	9943	62393015	62.38

**Table 5:** Detailed overview of frequent DNA copy number aberrations (>30%) of tumors from Caucasian SA patients.

chromosomal aberrations		flanking clones		position (bp)		segment size
gains	losses	start	end	start	end	(Mb)
	3p14.2	RP11-48E21	RP11-641C17	60380670	60705094	0.32
3q26.2-q26.31		RP11-669J9	RP11-44A1	172392313	173855790	1.46
	4q32.1-q35.2	RP11-192D11	CTC-963K6	159886665	191158370	31.27
5p13.1-p12		RP11-17J3	RP11-55O15	40113135	44396362	4.28
7p22.3-p21.3		RP11-713A20	RP11-505D17	106471	7932634	7.83
7q22.1		RP4-550A13	RP11-333G13	98512376	101153193	2.64
8p23.1		RP11-241P12	RP11-589N15	9788949	11803111	2.01
8q22.1-q22.3		RP11-664H21	RP11-132E3	98618965	105402542	6.78
8q24.21		RP11-28I2	RP11-1142f3	127563658	129620230	2.06
	9p24.1-p23	RP11-165O14	RP11-91E3	5873408	9689968	3.82
9q33.3-q34.3		RP11-62A6	RP11-424E7	124479347	138363252	13.88
11q13.3-q13.5		RP11-554A11	RP11-98G24	68509550	77008323	8.50
16p11.2		RP11-110P16	RP11-388M20	28675396	31163676	2.49
17q12-q21.1		RP5-986F12	RP11-94L15	33099924	35227135	2.13
17q25.1-q25.3		RP11-41E12	RP11-258N23	68729134	78451750	9.72
19p13.3-p13.11		RP11-110A24	RP11-88I12	134914	19023254	18.89
19q13.11-q13.43		CTC-1325L16	GS1-1129C9	37623641	63771717	26.15
20p13-q13.33		RP11-48M7	CTB-81F12	3728265	62393015	58.66

**Figure 3:**
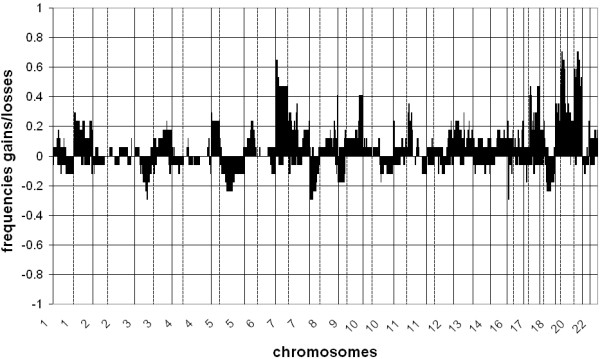
**Frequencies of gains and losses throughout the genome in all gastric adenocarcinomas from native SA patients**. Clones are sorted by position per chromosome (1-22). Vertical lines indicate transition between chromosomes; dashed vertical lines indicate centromere position.

**Figure 4:**
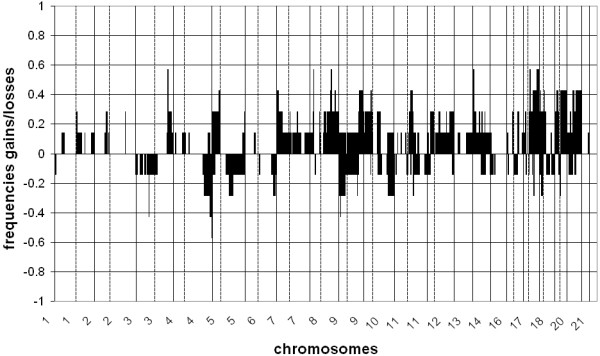
**Frequencies of gains and losses throughout the genome in all gastric adenocarcinomas from Caucasian SA patients**. Clones are sorted by position per chromosome (1-22). Vertical lines indicate transition between chromosomes; dashed vertical lines indicate centromere position.

### Supervised analysis

To identify biological differences between gastric cancers from different geographical origin, native SA tumors were compared with UK tumors using CGH test. Only MSS tumors were included in the supervised analysis. In total, 133 regions, located on different chromosomes, were significantly different (p < 0.05 and fdr≤0.15) between these two patient groups. An overview of the significant chromosomal regions, including the fdr rates, is given in Table 6. No significant differences were found between gastric cancers from UK and Caucasian SA patients or between gastric cancers from native and Caucasian SA patients.

**Table 6:** Detailed overview of the supervised analysis using CGH test.

cytoband	region start (bp)	region end (bp)	p-value	fdr	cytoband	region start (bp)	region end (bp)	p-value	fdr
1p36.33	672780	1359795	0.04	0.15	11p14.2-p14.1	27033269	27371257	0.05	0.15
1p36.32-p36.31	3386389	6294064	0.03	0.12	11q13.3	68509550	69323966	0.04	0.14
1p36.21-p36.13	12798944	15683816	0.03	0.12	11q13.3-q13.4	69314721	70472869	0.04	0.14
1p31.2	67178936	69303906	0.04	0.14	11q22.1-q22.2	98930611	101405228	0.03	0.12
1p31.1	69815162	76679895	0.01	0.06	11q22.2-q22.3	102010610	102424014	0.03	0.12
1p31.1	77428804	77820126	<0.01	0.04	12q21.2	76570565	78724263	0.04	0.14
1p21.2-p21.1	101684496	104502748	0.03	0.12	13q21.31-q21.33	61335626	69275204	0.04	0.14
1q31.1-q31.2	184717073	188976520	0.01	0.09	14q21.1	39694531	42171623	0.02	0.10
1q31.2-q31.3	189822405	193082884	0.02	0.10	14q21.2	42965408	44043547	0.01	0.09
1q31.3	193336091	194242465	0.01	0.08	14q21.2-q21.3	45258184	48298851	0.03	0.12
1q31.3	195068870	195629725	0.03	0.12	15q14	33841939	35953471	0.04	0.14
3p26.3	46140	2377366	<0.01	0.03	15q22.2	57373165	61214280	0.02	0.09
3p24.3	17181327	18148477	<0.01	0.03	15q23	65816865	68768615	0.02	0.09
3p24.3	19033520	21742232	<0.01	0.03	15q23-q24.2	69188639	73645336	0.05	0.15
3p24.3-p24.1	22747912	27531283	0.02	0.10	15q24.2-q25.2	74334873	81024206	0.03	0.12
3p21.31	46545403	47371983	0.05	0.15	16p13.2-p13.12	8777494	12522798	0.05	0.15
3p21.31	47384745	50114898	0.04	0.14	16p11.2	28675396	31163676	<0.01	0.04
3p21.31-p21.2	50533656	51418837	0.01	0.09	16q24.1	82993991	83622386	0.05	0.15
3p21.31-p21.2	51390596	52007218	0.02	0.09	16q24.1	84123856	84922042	0.02	0.09
3p21.1	52658450	53621497	0.01	0.09	16q24.2-q24.3	86986672	87452904	0.03	0.12
3p12.3	76450939	79097142	0.02	0.09	16q24.3	87848795	88465228	0.02	0.09
3p12.3-p12.2	79197544	82066233	<0.01	0.04	16q24.3	88398231	88613383	0.01	0.07
3p12.2-p12.1	82657794	84801874	<0.01	0.03	17p13.3	427024	1071560	<0.01	0.03
3p12.1-p11.2	85234579	87730259	<0.01	0.03	17p13.3-p13.2	2026966	4169913	0.04	0.14
3p11.1	88915283	89771786	<0.01	0.04	17p13.2	4810523	5166678	0.01	0.09
3q11.2	95569618	96250439	0.01	0.05	17p13.1	6780962	7477414	<0.01	0.03
3q25.1-q25.33	151508469	161536133	0.03	0.12	17p13.1	7436435	7682367	<0.01	0.03
3q25.33-q26.1	162044657	164516640	0.03	0.12	17p11.2	17343389	19251691	0.04	0.14
3q26.1	165289729	167697600	0.05	0.15	17q11.2	23133763	28423820	0.02	0.09
3q26.31-q26.32	174848631	180449332	0.05	0.15	17q11.2-q12	28664889	29147086	0.02	0.09
4p15.32-p14	17799729	36260048	0.02	0.11	17q25.1	69709369	71238958	0.01	0.09
4p14	36998587	37497326	0.01	0.09	17q25.1-q25.3	71406319	77588058	0.03	0.12
4p14	37851044	38103870	0.02	0.11	18q11.2	21431314	21774952	0.01	0.05
4p14-p12	38087293	46781512	0.01	0.09	18q11.2	22104619	22931012	<0.01	0.04
4q12	58332155	59148507	0.04	0.14	18q12.1	23970882	24526449	0.02	0.10
4q12-q13.1	59679465	62653308	0.02	0.10	18q12.1	25034674	26878315	0.01	0.08
4q13.3	74322497	76429795	0.03	0.12	18q12.1	27447009	28415095	0.01	0.09
4q13.3-q21.21	76495364	79353372	0.02	0.09	18q12.1	29409483	30219417	0.01	0.09
4q21.21	79222718	80683287	0.03	0.12	18q12.1-q12.2	30773824	31588529	0.01	0.06
4q33-q34.3	172094999	178721484	0.02	0.10	18q22.1	60340433	61310295	0.03	0.12
4q34.3	178969859	179599683	0.01	0.09	18q22.1	61623805	62645202	0.03	0.12
4q34.3	180819690	183096645	0.02	0.10	18q22.1-q22.3	63092764	68041625	0.03	0.12
4q35.1	184503994	186178296	0.03	0.12	19p13.3	134914	913289	0.01	0.08
5q11.2	50971745	52055659	0.04	0.14	19p13.3	902641	5009969	<0.01	0.04
5q11.2	52909242	56437163	0.03	0.12	19p13.3	5663923	6519297	<0.01	0.01
5q11.2-q12.1	56921490	58947885	0.02	0.09	19p13.3-p13.2	6523443	9826740	<0.01	<0.01
5q14.3	82802677	86118543	0.05	0.15	19p13.2-p13.12	10248852	15116365	<0.01	0.03
5q23.2	122463056	123527915	0.05	0.15	19p13.12-p13.11	15415833	17777501	<0.01	0.01
7p22.1-p21.3	6983150	7932634	<0.01	0.02	19p13.11	18202507	19023254	<0.01	0.03
7p21.3-p21.2	9256088	14085902	<0.01	<0.01	19p12	19877150	21504328	0.01	0.05
7p21.2-p21.1	14342152	19957111	<0.01	0.01	19p12	22133662	22949959	0.01	0.05
7q22.1	98651132	100929260	<0.01	0.03	19q12	33315121	33507712	0.02	0.10
8q24.21	130562467	131126185	0.05	0.15	19q12	34159370	34664148	0.02	0.11
8q24.22	131641447	133561490	0.01	0.09	19q12	34960906	35766560	0.02	0.11
8q24.22	134537164	136154996	0.02	0.11	19q12-q13.11	36958851	37518517	0.05	0.15
8q24.22-q24.23	136472354	138543270	0.04	0.14	19q13.12-q13.13	40883472	43004503	0.03	0.13
8q24.3	141395868	142790557	0.01	0.05	19q13.2-q13.32	46498596	50725496	0.03	0.12
8q24.3	144790054	145357620	0.01	0.09	19q13.32-q13.33	52665374	54718281	0.03	0.12
8q24.3	145585590	145953950	0.02	0.11	19q13.33-q13.43	55461670	63771717	<0.01	0.01
8q24.3	145893230	146238749	0.04	0.14	21q11.2-q21.1	13857799	18774434	<0.01	0.03
9p24.1-p23	8398601	9689968	0.04	0.14	21q21.1	20982315	21411332	<0.01	0.03
9p23	9684353	10554235	0.04	0.14	21q21.1-q21.2	22151920	22943367	<0.01	0.03
10q22.1	70824040	71674097	0.03	0.12	21q21.2	23491399	25568510	<0.01	0.01
10q22.1	72033907	73573433	0.02	0.09	21q21.3	26174732	26923374	0.02	0.09
10q26.3	135110821	135301208	0.03	0.12	21q21.3-q22.11	28596969	30855176	0.03	0.12
11p15.5	178227	626401	0.01	0.05	22q13.33	48473404	49397088	0.04	0.15
11p15.5	1299306	1785278	<0.01	0.03					

In total, 133 regions were significantly different (p < 0.05 and fdr≤0.15) between UK and native SA tumors. The chromosomal regions, including the start and end positions, and the fdr rates are listed.

## Discussion

One of the main risk factors contributing to gastric cancer is infection with *H. pylori*, which causes a chronic active gastritis [4,21]. In South Africa, gastric cancer is infrequent, while the prevalence of *H. pylori *infection is very high. Although differences in genotypes of *H. pylori *exist in different geographic areas, this African enigma can not only be explained by differences in virulent strains of *H. pylori *[22-24]. High prevalence of *vac*A s1b strain is observed in South Africa as well as in Brazil and Portugal, countries with high incidences of gastric cancer,[25-27] and frequencies of *CagA *antibodies were similar between patients with gastric neoplasia compared to healthy controls [28]. The prevalence of the different virulent strains in the present study is unknown. Since *H. pylori *is thought to play a major role in the initiation phase of gastric cancer development and most often already disappeared at time of gastric cancer diagnosis, it is impossible to accurately retrieve this information.

Besides the virulence of the infecting *H. pylori *strain, other factors influence gastric cancer risk, including environmental factors such as diet and socioeconomic status, and host factors, such as polymorphisms, which are involved in the inflammatory response to the infection [29,30]. Knowing that the prevalence of *H. pylori *infection and incidences of gastric cancer are different in South Africa and Western Europe, we aimed to study if this would reflect in different patterns of gastric carcinogenesis.

The concept of the African enigma has been challenged since it has been suggested that the enigma could be explained due to lack of infrastructure and access to hospitals and care in African countries resulting in incomplete reporting of gastric cancer. However the incidence of gastric cancer would have been so dramatically underestimated that it has been stated that under-reporting by itself could not explain the lower frequencies of gastric cancer in African countries [31]. Also, when using the proportional frequency of gastric cancer compared to other cancer types in Africa, gastric cancer incidence remains very low [8]. Another criticism on the African enigma has been the high prevalence of HIV infection. A relatively large part of the African population would die of HIV before the age in which gastric cancer becomes more frequent. However, the low gastric cancer incidence in Africa was described before the HIV epidemic.

South African patients showed significantly more microsatellite instable gastric cancers compared to Western European patients. Also at the level of chromosomal instability clear differences were found, reflected by a significant correlation between cluster membership and geographical tumor origin, i.e. UK, native SA and Caucasian SA. Microsatellite instable gastric cancers are described to have fewer chromosomal aberrations compared to microsatellite stable gastric cancers [32,33]. To rule out that tumors from South African patients cluster together by hierarchical cluster analysis due to the fact that these tumors show higher frequencies of microsatellite instability, only microsatellite stable gastric cancers were included in the hierarchical cluster analysis.

Not much has been reported about microsatellite status in gastric cancers from African patients. One study reported infrequent microsatellite instable gastric cancers in South African patients[34] which is in contrast with our findings which show a higher frequency of microsatellite instability in gastric cancers from SA patients compared to UK patients. Based on the present data, MSI does play an important role in gastric carcinogenesis in South Africa.

Several chromosomal aberrations are common in the three different tumor groups analyzed, including gains of chromosomes 7, 8q, 9q, 17q, 19 and 20 and losses of 3p and 4q, while other chromosomal changes are specific for each tumor group. In addition, gastric cancers from UK patients showed a significantly higher number of clones showing a loss compared to gastric cancers form South African patients. These results indicate different patterns of chromosomal instabilities in gastric cancers correlating to geographical origin of the patient.

The chromosomal aberrations of the UK tumors are comparable to other array CGH studies analyzing Western European tumors [12,35-37]. To the best of our knowledge, this is the first array CGH study on gastric cancers from South African patients. Since several chromosomal regions are significantly different between gastric cancers from different geographical origin, and each region comprises multiple genes, further studies are needed to pinpoint candidate genes contributing to the differences in genomic profiles.

The higher frequency of diffuse gastric cancers from UK patients compared to the SA patients in the present study could be considered as a confounding factor. Contradicting results have been published either describing different or similar patterns of DNA copy number aberrations between intestinal and diffuse type gastric cancers [7,33,37-39]. In the context of the present study we believe that the differences in DNA copy number aberrations between UK and SA gastric cancers are independent of the histological tumor type. When repeating the cluster analysis with intestinal type carcinomas only, cluster membership again was significantly correlated with geographical origin of the tumors. Also supervised data analysis, i.e. testing copy number status of all genomic loci, did not reveal any significant differences in DNA copy number changes between intestinal and diffuse gastric cancers from UK patients. Furthermore, hierarchical cluster analysis including UK gastric cancers only did not separate intestinal and diffuse type gastric cancers. We therefore do not believe our findings to be influenced by distribution of histological types in this series. Question remains why diffuse type gastric cancers were more frequently observed in gastric cancers from UK patients compared to SA patients. Besides being a confounding factor, we can hypothesize that mutation of E-cadherin (*CDH1*), or other mechanisms disrupting the *CDH1 *gene function such as epigenetic mechanisms or miRNAs, playing an important role in diffuse type gastric cancer, might play a minor role in SA gastric cancer patients due to different pathways of carcinogenesis, as shown in the present study by differences in patterns of DNA copy number aberrations. Also, the prevalence of *H. pylori *infection is very high in South Africa, and *H. pylori *infection mainly plays a role in intestinal type gastric cancers. This could also explain the higher number of intestinal type gastric cancers in SA patients.

Further, with respect to copy number changes in relation to histological types, chromosomal gains of 8q and 17q and losses of 3p have been described to be associated with intestinal type gastric cancers [33]. On the other hand, gains of 8q and 17q have been reported to be altered predominantly in diffuse type gastric cancers [38]. In the present study gains on chromosomes 8q and 17q and losses on 3p were common to both intestinal and diffuse type gastric cancers. In addition, these aberrations also were common in tumors from both UK and SA patients. Gains on chromosomes 13q and 19q have been found more frequently in diffuse type gastric cancers [33,36,38]. Again, in the present study, gains of these chromosomes were observed equally in intestinal and diffuse type gastric cancers. Gain of 19q was frequently observed in tumors from both geographical origins. Although gain of 13q was observed less frequently in tumors from SA patients compared to tumors from UK patients, still around 20-25% of the tumors of native SA patients show a gain of chromosome 13q, making it unlikely that tumor type has influenced cluster membership.

A limitation of the present study is the fact that native SA gastric cancer patients were significantly younger compared to Caucasian SA and UK gastric cancer patients. We previously showed that gastric cancers of young and elderly patients have different patterns of chromosomal aberrations [12]. We cannot rule out that also in these series, age might contribute to differences in DNA copy number profiles, however cluster analysis showed that gastric cancers from native SA patients were more similar to cancers from Caucasian SA patients, who have similar age as UK patients, indicating that cluster membership is independent of age in this respect. Overall, most differences were observed between UK and native SA tumors.

We realize that the present study is based on a heterogeneous group of gastric cancer patients, with different genetic background and different environmental factors, including *H. pylori*, diet and socioeconomic status, influencing gastric cancer risk. Statements on genotype influencing gastric cancer are very difficult to make since the degree of heterogeneity within each different patient group, i.e. UK, native SA and Caucasian SA, is unknown.

The patterns of genomic alterations in gastric cancers from UK and SA patients could gain clinical relevance in the future. In addition to surgery, gastric cancer treatment increasingly includes (neo)adjuvant chemotherapy and/or radiotherapy, however still without patients being stratified based on biological characteristics of their tumors. Clinical trials are underway in which also the value of genetic markers for predicting response to therapy are studied. In the end, stratification for therapy may include genomic alterations observed in tumors of patients from different geographical origin.

## Conclusions

We showed that gastric cancers of UK and SA patients are different in their patterns of genomic instability. Gastric cancers from SA patients show higher frequencies of microsatellite instability and different patterns of chromosomal aberrations compared to gastric cancers from UK patients. These results may suggest different molecular pathways of gastric carcinogenesis, consistent with the African enigma hypothesis. Further studies are needed to explore the link between *H. pylori *and other environmental factors, as well as host factors, such as polymorphisms influencing gastric cancer susceptibility, in relation to the patterns of genomic instability in gastric cancers from these different geographic areas.

## Competing interests

The authors declare that there are no competing interests.

## Authors' contributions

TB performed part of the DNA isolations, array CGH experiments and MSI analysis, performed data analysis and wrote the manuscript, ML collected the material from South African patients, performed part of the DNA isolations and array CGH experiments and helped in conceiving the study. NG revised the carcinomas included in the study and helped in coordinating the study and writing of the manuscript, MT helped in performing array CGH experiments and MSI analysis, BC helped in coordinating the study, BY provided the facilities for the microarray experiments, HG provided the material from the United Kingdom, CM, CV, and SM were involved in conceiving and coordinating the study, GM was involved in coordinating the study and writing of the manuscript. All authors read and approved the final version of the manuscript.

## Pre-publication history

The pre-publication history for this paper can be accessed here:

http://www.biomedcentral.com/1755-8794/4/7/prepub
